# WS-SNPs&GO: a web server for predicting the deleterious effect of human protein variants using functional annotation

**DOI:** 10.1186/1471-2164-14-S3-S6

**Published:** 2013-05-28

**Authors:** Emidio Capriotti, Remo Calabrese, Piero Fariselli, Pier Luigi Martelli, Russ B Altman, Rita Casadio

**Affiliations:** 1 Division of Informatics, Department of Pathology, University of Alabama at Birmingham, Birmingham AL, USA; 2 S-IN Soluzioni Informatiche Srl, Vicenza, 36100, Italy; 3 Department of Computer Science, University of Bologna, Bologna, 40126, Italy; 4 Laboratory of Biocomputing, Department of Biology, University of Bologna, Bologna, 40126, Italy; 5 Departments of Bioengineering and Genetics, Stanford University, Stanford, CA, USA

## Abstract

**Background:**

SNPs&GO is a method for the prediction of deleterious Single Amino acid Polymorphisms (SAPs) using protein functional annotation. In this work, we present the web server implementation of SNPs&GO (WS-SNPs&GO). The server is based on Support Vector Machines (SVM) and for a given protein, its input comprises: the sequence and/or its three-dimensional structure (when available), a set of target variations and its functional Gene Ontology (GO) terms. The output of the server provides, for each protein variation, the probabilities to be associated to human diseases.

**Results:**

The server consists of two main components, including updated versions of the sequence-based SNPs&GO (recently scored as one of the best algorithms for predicting deleterious SAPs) and of the structure-based SNPs&GO^3d ^programs. Sequence and structure based algorithms are extensively tested on a large set of annotated variations extracted from the SwissVar database. Selecting a balanced dataset with more than 38,000 SAPs, the sequence-based approach achieves 81% overall accuracy, 0.61 correlation coefficient and an Area Under the Curve (AUC) of the Receiver Operating Characteristic (ROC) curve of 0.88. For the subset of ~6,600 variations mapped on protein structures available at the Protein Data Bank (PDB), the structure-based method scores with 84% overall accuracy, 0.68 correlation coefficient, and 0.91 AUC. When tested on a new blind set of variations, the results of the server are 79% and 83% overall accuracy for the sequence-based and structure-based inputs, respectively.

**Conclusions:**

WS-SNPs&GO is a valuable tool that includes in a unique framework information derived from protein sequence, structure, evolutionary profile, and protein function. WS-SNPs&GO is freely available at http://snps.biofold.org/snps-and-go.

## Background

In the last few years the cost of high-throughput sequencing experiments has rapidly decreased; however the analysis and interpretation of sequencing data are still challenging issues in Molecular Biology and Bioinformatics [1]. During the last 10 years of experiments, over 3,000 billions of nucleotides from human genomes have been released [2]. This information in conjunction with new data from the HapMap Consortium [3] and the Human Variation Project [4] allowed to identify tens of millions of Single Nucleotide Polymorphisms (SNPs) as the main cause of variability between individuals [5]. Currently dbSNP [6], which is the most comprehensive database of genetic variations, collects ~51.8 million of SNPs. Depending on the region where they occur, SNPs could affect gene expression and function with different mechanisms [7]. Although recent published data from the ENCODE Consortium [8] enabled to assign a biochemical function to the 80% of non-coding regions, the non-synonymous variations in the coding regions still represent the largest component of genetic variants annotated as *"pathogenic" *in the dbSNP database. For this reason, the annotation of non-synonymous SNPs (nsSNPs) is important to understand the relationships between variations and diseases.

Curators of dbSNPs [6] and SwissVar [9] databases are collecting information to annotate the impact of SNPs on human health. The process requires expensive and time-consuming functional experiments and clinical trials. Thus, during the last few years, several methods have been developed to predict the impact of nsSNPs on protein stability [10-16], protein function [17,18] and to detect their pathologic effect [19-29]. A more comprehensive lists of these tools is available on a recent review [30]. In particular, the class of algorithms, capable of discriminating between disease-related and neutral SNPs can be extensively used for personal genome interpretation [30] and personalized medicine [1]. These methods are mainly based on statistical and/or machine learning approaches that take as input information from protein sequence [18,19,23,24,26,28,29], structure [20,25] and function [19,21,27].

In general, all the predictors rely on evolutionary information that is extracted using different procedures. For example, the SIFT algorithm [18] exploits the information contained in sequence alignments to calculate the probability that a mutation of a residue in a given sequence position is tolerated. For instance, if a position in an alignment contains hydrophobic residues, then SIFT assumes that the position can only contain hydrophobic residues. PhD-SNP is a machine learning approach that takes as input the frequencies of wild-type and mutant residues from a sequence profile calculated with the BLAST algorithm [31]. PolyPhen [25] evaluates a substitution score calculating the Position Specific Independent Count (PSIC) matrix. More sophisticated methods such as MutPred [24] and SNPs&GO [19] also include outputs of other predictors and/or a functional score calculated using the Gene Ontology [32]. In this paper we present the web server implementation of the SNPs&GO algorithms (based on sequence or structure inputs). The server is freely available to the whole scientific community.

## Methods

### Dataset and benchmarking

Both the sequence and structure based algorithms have been trained on a set of disease-related and neutral variations from the SwissVar database (October 2009). In details, the sequence-based method has been trained using a 20-fold cross-validation procedure on a set of 38,460 single point variations from 9,067 proteins (SAP-SEQ). The structure-based algorithm has been trained on the subset of 6,630 variations that map on 721 protein chains (SAP-3D) with available structure in the Protein Data Bank (PDB) [33]. The SAP-3D dataset has been obtained mapping the SwissVar variants on the PDB according to the procedure previously described [20]. Both SAP-SEQ and SAP-3D datasets are composed approximately by the same fraction of disease-related and neutral variants (Table 1). In the SAP-3D dataset, to balance the fraction of disease related to that of neutral variants, we increased the number of neutral variants assuming that the reverse of a neutral variant is also neutral. We also performed a blind test on an independent data set of protein variations annotated in SwissVar from October 2009 to December 2011 and with resolved protein structure available in the PDB (SAP-NEW). The SAP-NEW is a unique dataset for testing sequence and structure based methods, that consists of 1,489 single variations from 271 proteins not included in the previous dataset. All the variations in SAP-NEW have been mapped on 290 PDB structures. All the datasets used in this paper are made available as supplementary files.

**Table 1:** Composition of the datasets

	SAP-SEQ	SAP-3D	SAP-NEW
Total variations	38,460	6,630	1,489
Disease related variations	19,230	3,342	960
Neutral variations	19,230	3,288	529
Proteins	9,067	721*	271

*protein sequences of SAP-SEQ, endowed with structure, that are used to train/test SNPs&GO^3d^.

### Measures of performance

In this work the efficiency of our predictors have been scored using the following statistical indexes.

The overall accuracy is:

(1) $$Q_{tot}=\frac{C}{T}$$

Where *C *is the total number of correctly predicted variations and *T *is the total number of variations. The Matthew's correlation coefficient *MCC *is defined as:

(2) $$MCC\left(s\right)=\frac{p\left(s\right)n\left(s\right)-u\left(s\right)o\left(s\right)}{W}$$

where *W *is the normalization factor:

(3) $$W=\sqrt{\left[\left(p\left(s\right)+u\left(s\right)\right)\left(p\left(s\right)+o\left(s\right)\right)\left(n\left(s\right)+u\left(s\right)\right)\left(n\left(s\right)+o\left(s\right)\right)\right]}$$

for each class *s *(D and N, disease-related and neutral variations respectively); *p(s) *and *n(s*) are the total number of correct predictions and correctly rejected assignments, respectively. *u(s) *and *o(s) *are the numbers of false negatives and false positives for the *s *class.

The coverage *S *(sensitivity) for each discriminated class *s *is evaluated as:

(4) $$S\left(s\right)=\frac{p\left(s\right)}{p\left(s\right)+u\left(s\right)}$$

where p(s) and u(s) are the same as in Equation 3.

The probability of correct predictions *P *(or positive predictive values or specificity) is computed as:

(5) $$P\left(s\right)=\frac{p\left(s\right)}{p\left(s\right)+o\left(s\right)}$$

Where *p(s) *and *o(s) *are the same as in Equation 3 (ranging from 0 to 1).

For each prediction a reliability score (*RI*) is calculated as follows:

(6) $$RI=20\times abs\left[O\left(D\right)-0.5\right]$$

where *O(D) *is the probability associated to the disease-related (D). O(D) is the output of the method (ranging from 0 to 1) returned when LIBSVM tool [34] is executed using the probability estimation option.

Finally the area under the ROC curve (AUC) is calculated by plotting the true positive rate (TPR = S(D)) as a function of the False Positive Rate (FPR = 1-P(N)) at different prediction thresholds.

### WS-SNPs&GO description

The SNPs&GO algorithms predict the impact of protein variations using functional information codified by Gene Ontology (GO) terms of the three main roots: Molecular Function, Biological Process and Cellular Component. Here we introduce a web server implementation of previous method, relying either on protein sequence and protein structure information, namely SNPs&GO and SNPs&GO^3d^, respectively. With respect to the previous version, the new SNPs&GO has slightly different input features representing the PANTHER output and the functional information. When compared with the standard sequence-based algorithm, in the recently developed SNPs&GO^3d ^[22], the sequence features were replaced with structural based features including the structure-environment and the solvent exposure of the mutated residue.

**Figure 1:**
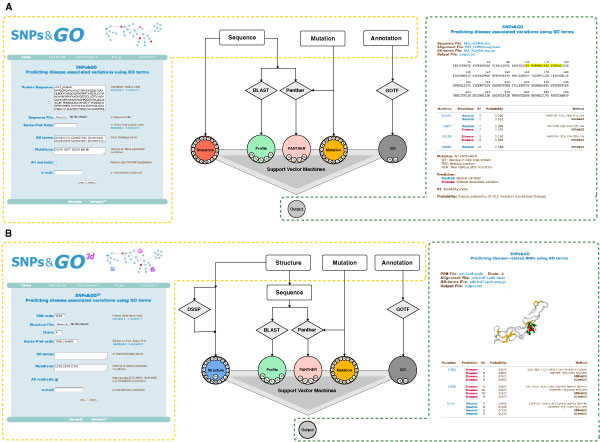
**Schematic view of SNPs&GO (panel A) and SNPs&GO^3d ^(panel B)**. From the left to the right, the SNPs&GO and SNPs&GO^3d ^input web pages, the flow chart of the sequence and structure-based methods and two examples of the returned outputs.

In Figure 1 we report a schematic view of the two Support Vector Machine (SVM) based classifiers able to discriminate between disease-related and neutral variations.

The SNPs&GO algorithm (Figure 1 panel A) takes in input only protein sequence information. For each given sequence, the algorithm automatically generates the input profile by calculating the pair-wise alignments with the BLAST algorithm [31]. The sequence profile is calculated performing one run of BLAST against the UniRef90 dataset (ftp://ftp.ebi.ac.uk/pub/databases/uniprot/uniref/) to select homologous sequences with E-value lower than 10^-9^. Besides features derived from sequence profile, the SVM input vector also includes the sequence environment of the variation and a log-odd score calculated considering all the Gene Ontology terms associated to the mutated protein and their parents in the GO graph. The SNPs&GO^3d ^algorithm (Figure 1 panel B) takes in input structural information and generates a SVM input vector where the sequence environment used in SNPs&GO, is replaced by the structural environment and the Relative Solvent Accessible Area (RSA) of the wild-type residue. To summarize, the sequence-based algorithm calculates for each mutation a 51-elements feature vector including: i) the mutation (20 values all set to 0 with the exception of the position corresponding to the mutated and wild-type residues that are set to 1 and -1, respectively), ii) the sequence environment (20 values corresponding to the frequency of the different residues in a 19-residue long window); iii) the sequence profile (5 values corresponding to: the elements of the profile related to the mutated and wild-type residues, the number of aligned sequences observed in the mutated position in the whole alignment and the conservation index of the mutated position [35]); iv) 4 elements features from the output of PANTHER algorithm [26] encoding for the probability of deleterious variations, the frequencies of wild-type and mutant residues, and the number of independent counts; v) the functional annotation score (2 values, the GO log-odd scores and the number of GO-terms used).

When for a given protein the structure is available, it is possible to run SNPs&GO^3d^. In this case, the server calculates a 52-elements feature vector where the 20-elements vector encoding for the sequence environment is replaced by a 20 elements vector encoding for the structural environment. The structural environment is computed considering the residue composition within a 6 Å radius sphere around the Cα (carbon alpha) of the wild-type residue. One further element is added to encode for RSA as derived from the DSSP program [36]. The remaining 31 input features are computed as described above for the sequence-based algorithm. With respect to a previous implementation (SNPs&GO [19]) the input vector of the sequence-based method differs by the bit indicating the presence of absence of the GO-terms (see [19]) that is now replaced by an integer value counting the number of GO-terms used to compute the GO-score (introduced already in [20]). In the case in which PANTHER algorithm is not able to return an output, an arbitrarily input vector is included assigning a probability of 0.5 for deleterious variations and 0 for the other three remaining PANTHER features. According to this choice, in the last version of SNPs&GO, we removed from the previous 5-elements feature vector the element indicating the presence of PANTHER output.

### WS-SNPs&GO input

Depending on the information available to the user, either SNPs&GO and/or SNPs&GO^3d ^can be activated. The server is endowed with two alternative input pages that are linked to the WS-SNPs&GO home page.

SNPs&GO input. The standard SNPs&GO server needs as input the protein sequence, its relative variations and the functional annotations (see Figure 1 panel A). The input can be provided in three different ways: i) by pasting in the appropriate textbox area the protein sequence in FASTA or raw format; ii) by uploading a file from the local machine; iii) by typing the SwissProt code. When the SwissProt code of the protein is provided, the server automatically assigns the associated GO terms of all the three subontologies (Biological Process, Cellular Component and Molecular Function) as defined in the Gene Ontology. Alternatively, protein functional annotation can be provided using the appropriate input box. In this case the server automatically runs the GO-TermFinder program [37] for the retrieval of all the GO-term ancestors. When functional information is not provided the method assigns zeros to the two-elements vector encoding the protein function.

SNPs&GO^3d ^input. The SNPs&GO^3d ^interface (see Figure 1 panel B) is slightly different because in this case the server requires structural information. The input consists of: i) the PDB code (or a PDB file) of the mutated protein and the relative chain; ii) the list of mutations, iii) the protein GO terms. Also in this case, when the SwissProt code of the mutated protein is provided, the server automatically assigns all the annotation terms. More details about the input features are described in a previous work [20].

### WS-SNPs&GO output

The server has been designed to return the prediction output on the fly, providing a link to a web page that is refreshed approximately each 20 seconds or by e-mail. The outputs of SNPs&GO and SNPs&GO^3d ^are similar. The html output page provides the links to the sequence or structure given in input, to the results of the BLAST search visualized with MView [38], to the file with all the GO terms associated to the mutated protein and the output in text format. In the second part of the output, the protein sequence is visualized and a table including all the mutations and their relative predictions is reported. In details, the table is composed of 5 columns, including the mutated residue, the prediction (either Disease or Neutral), the reliability index (RI), the probability associated to the disease-related class and the information about the prediction method. If the probability corresponding to disease-related is larger than 0.5 the variation is predicted as disease-related. In addition, a click on the variations in the output table, allows to highlight the mutated residue in the protein sequence visualized above. When available, the server also reports the output of the PANTHER algorithm [26] which is included in the input features of SNPs&GO (see WS-SNPs&GO, Description section). When the protein function is not available, the "All methods" option runs PhD-SNP [19] and S3D-PROF (the 3D structure version of PHD-SNP). Both programs are based on sequence or structure profiles and the mutation environment. For SNPs&GO^3d ^the server returns outputs similar to those of SNPs&GO. The output includes also the Relative Solvent Accessible area (RSA) of the mutated residue calculated using the DSSP program [36]. In the case of structural prediction the server exploits Jmol applet (http://sourceforge.net/projects/jsmol/) to visualize the protein structure and a click on the variation shows the mutated residue (in red) and its structural environment (in green). When the "All methods" option is activated the SNPs&GO^3d ^algorithm also returns the standard sequence-based SNPs&GO prediction.

## Results

When tested using a cross-validation procedure where variations in similar proteins are kept in the same subset (as determined with *blastclust*, by setting 30% sequence identity and 80% coverage), the revised SNPs&GO and SNPs&GO^3d ^reach respectively 81% and 84% overall accuracies, 0.61 and 0.68 Matthews correlation coefficients (MCCs) and 0.89 and 0.91 AUCs of the ROC curve. The performances of both methods are listed in Table 1. The performance of the sequence-based method is not significantly affected when the dataset is reduced (SAP-SEQ vs SAP-3D) as indicated in Table 1. In the Tables 2 and 3, we also report the scores of different implementations taking less input features (PhD-SNP, S3D-PROF) on SAP-SEQ and SAP-3D datasets.

**Table 2:** Performance of the different methods on the SAP-SEQ dataset

Method	Q_tot_	P[D]	S[D]	P[N]	S[N]	MCC	AUC
PhD-SNP	0.76	0.77	0.75	0.76	0.78	0.52	0.83
SNPs&GO	0.81	0.82	0.79	0.80	0.82	0.61	0.88

D=disease related, N=neutral. For index definition see "Measures of performance".

**Table 3:** Performance of the different methods on the SAP-3D dataset

Method	O_tot_	P[D]	S[D]	P[N]	S[N]	MCC	AUC
PhD-SNP	0.79	0.83	0.74	0.76	0.84	0.58	0.87
S3D-PROF	0.81	0.80	0.84	0.83	0.78	0.63	0.88
SNPs&GO	0.81	0.80	0.83	0.82	0.78	0.61	0.89
SNPs&GO^3d^	0.84	0.82	0.86	0.85	0.81	0.68	0.91

D=disease related, N=neutral. For index definition see "Measures of performance".

**Figure 2:**
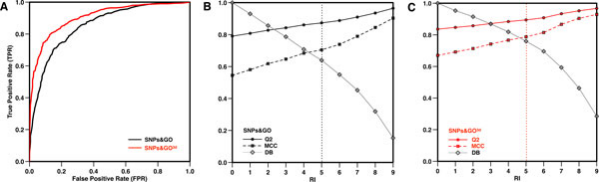
**Performance of SNP&GO and SNPs&GO^3d ^on the SAP-NEW dataset (DB)**. In panel A the ROC curves of both methods are shown. In panels B and C the performances of SNP&GO and SNPs&GO^3d ^as a function of the Reliability index (RI) are reported.

The methods have been also tested on a set of variants recently annotated in SwissVar database. In order to properly assess the method performance, we predict the variations of SAP-NEW using SVM models whose training set contains only sequences with less than 30% identity and 80% of coverage with the one to be predicted. On this set the sequence based version (SNPs&GO) scores with 79% overall accuracy, 0.54 MCC and 0.85 AUC and the structure-based version (SNPs&GO^3d^) scores with 83% overall accuracy, 0.67 MCC and 0.91 AUC (see Table 4 and Figure 2 panel A). Using the output of our SVM-based method we calculate the reliability index (RI) associated to the prediction (for RI definition see the Method section). RI values are useful to select more accurate predictions. For example, if we select predictions with a reliability index RI≥5, SNPs&GO and SNPs&GO^3d ^reach respectively 87% and 89% overall accuracy, 0.70 and 0.79 MCC over ~64% and 76% of the SAP-SEQ and SAP-3D datasets (see Figure 2 panels B and C). PhD-SNP and S3D-PROF are also evaluated over the SAP-NEW dataset (see Table 4).

**Table 4:** Performance of the different methods on the SAP-NEW dataset

Method	Q_tot_	P[D]	S[D]	P[N]	S[N]	MCC	AUC
PhD-SNP	0.70	0.77	0.75	0.57	0.61	0.35	0.74
S3D-PROF	0.80	0.80	0.84	0.80	0.76	0.60	0.87
SNPs&GO	0.79	0.84	0.83	0.71	0.71	0.54	0.85
SNPs&GO^3d^	0.83	0.84	0.86	0.84	0.80	0.67	0.91

D=disease related, N=neutral. For index definition see "Measures of performance

It has to be noticed that the accuracies of the sequence-based methods (SNPs&GO and PhD-SNP) on the SAP-NEW dataset are lower than those obtained on SAP-SEQ dataset. This difference can be due to the possible fluctuations that strongly affect small datasets. Indeed, poorer SNPs&GO and PhD-SNP predictions are mainly observed in the subset of SNP-NEW neutral polymorphisms that is composed by only 529 mutations that correspond to ~3% of those in SAP-SEQ. The comparison of the results obtained by S3D-PROF and SNPs&GO^3d ^on SAP-3D and SAP-NEW datasets shows that structural information allows to partially recover the loss accuracy due to less discriminative sequence based features. This observation reinforces the idea that protein structure is an important piece of information to improve the detection of disease-related variants.

## Conclusions and discussion

Recently it has been observed that the correlation among disease associated variation types and perturbation of protein stability is moderate [39]. The advantage of the WS-SNPs&GO server is that the impact of SAPs is predicted directly from variations in the protein sequence and/or structure relying on function. When the GO-score computation does not require the reconstruction of ancestry paths in the GO graph (20), SNPs&GO returns its prediction in a time interval comparable with one run of the BLAST algorithm on the UniRef90 database. Our server is a good alternative to well-established tools like SIFT [18] and PolyPhen [23] since it returns high quality predictions as shown in previous works [19,20,40] and confirmed in the 2011 edition of the Critical Assessment of Genome Interpretation experiments (http://genomeinterpretation.org/). In particular SNPs&GO was scored among the best methods in the prediction of deleterious mutations in RAD50. To our knowledge WS-SNPs&GO is the only function-based server for the prediction of deleterious variants tested on a large number of single point variations related to all types of disease. Finally, it is also worth noticing that the sequence-based method SNPs&GO in its previous version [19] has been scored among the best methods for the prediction of deleterious protein variations by independent assessors [40]. Here we present an updated version together with SNPs&GO^3d ^that can exploit (when available) the structural information of proteins and when this is possible, it returns more accurate results. We propose our WS-SNPs&GO server as a useful source of annotation of protein variations in transcript and exome sequencing high-throughput experiments.

## Competing interests

The authors declare they have no conflict of interests in relation to the SNP-SIG issue article.

## Authors' contributions

EC and RCalabrese developed the method and the web server. EC, PF, RBA and RCasadio conceived this work and participated in design and coordination. EC, PF, PLM and RCasadio wrote the manuscript. All authors read and approved the manuscript.

## Additional file 1

SAP-SEQ dataset: http://snps.biofold.org/snps-and-go/pages/data/200910_SAP-SEQ.txt

## Additional file 2

SAP-3D dataset: http://snps.biofold.org/snps-and-go/pages/data/200910_SAP-3D.txt

## Additional file 3

SAP-NEW dataset: http://snps.biofold.org/snps-and-go/pages/data/201112_SAP-NEW.txt

## Declarations

The publication costs for this article were funded by the above grants supporting EC and RCasadio.

This article has been published as part of *BMC Genomics *Volume 14 Supplement 3, 2013: SNP-SIG 2012: Identification and annotation of SNPs in the context of structure, function, and disease. The full contents of the supplement are available online at http://www.biomedcentral.com/bmcgenomics/supplements/14/S3
